# A quantitative comparison of the performance of three deformable registration algorithms in radiotherapy

**DOI:** 10.1016/j.zemedi.2013.07.006

**Published:** 2013-12

**Authors:** Daniella Fabri, Valentina Zambrano, Amon Bhatia, Hugo Furtado, Helmar Bergmann, Markus Stock, Christoph Bloch, Carola Lütgendorf-Caucig, Supriyanto Pawiro, Dietmar Georg, Wolfgang Birkfellner, Michael Figl

**Affiliations:** 1 Center of Medical Physics and Biomedical Engineering, Medical University of Vienna, AKH-4L, Waehringer Guertel 18-20, A-1090 Vienna, Austria; 2 Department of Radiotherapy, Division of Medical Radiation Physics, Medical University of Vienna, Waehringer Guertel 18-20, AKH, A-1090 Vienna, Austria; 3 Christian Doppler Laboratory for Medical Radiation Research for Radiation Oncology, Medical University of Vienna, Waehringer Guertel 18-20, AKH, A-1090 Vienna, Austria

**Keywords:** Deformable registration, radiotherapy, organ motion, Deformierbare Registrierung, Radiotherapie, Organbewegung

## Abstract

We present an evaluation of various non-rigid registration algorithms for the purpose of compensating interfractional motion of the target volume and organs at risk areas when acquiring CBCT image data prior to irradiation. Three different deformable registration (DR) methods were used: the Demons algorithm implemented in the *iPlan* Software (BrainLAB AG, Feldkirchen, Germany) and two custom-developed piecewise methods using either a Normalized Correlation or a Mutual Information metric (featurelet_*NC*_ and featurelet_*MI*_). These methods were tested on data acquired using a novel purpose-built phantom for deformable registration and clinical CT/CBCT data of prostate and lung cancer patients. The Dice similarity coefficient (DSC) between manually drawn contours and the contours generated by a derived deformation field of the structures in question was compared to the result obtained with rigid registration (RR). For the phantom, the piecewise methods were slightly superior, the featurelet_*NC*_ for the intramodality and the featurelet_*MI*_ for the intermodality registrations. For the prostate cases in less than 50% of the images studied the DSC was improved over RR. Deformable registration methods improved the outcome over a rigid registration for lung cases and in the phantom study, but not in a significant way for the prostate study. A significantly superior deformation method could not be identified.

## Zusammenfassung

In vorliegender Arbeit wird eine Evaluierung verschiedener nicht-rigider Registrationsalgorithmen zur Kompensation interfraktioneller Bewegungen des Zielvolumens und von Risikoorganen anhand von vor der Bestrahlung gewonnenen Conebeam-Computertomographien (CBCT) vorgestellt. Drei verschiedene Methoden zur deformierbaren Registrierung (DR) kamen hierbei zur Anwendung: Einerseits wurde der Demons-Algorithmus der *iPlan* Software (BrainLAB AG, Feldkirchen, Deutschland) verwendet, andererseits kamen zwei Eigenentwicklungen zur stückweise rigiden Registrierung zum Einsatz. Letztere verwendeten entweder eine normierte Korrelationsmetrik (featurelet_*NC*_) oder eine auf der Mutual Information basierende Bildvergleichsmethode (featurelet_*MI*_). Diese Verfahren wurden mit einem neuartigen Phantom für die *DR*- und klinischen *CT*- bzw. *CBCT-Daten* von Prostata- und Lungenkarzinompatienten validiert. Die Ergebnisse wurden anhand des *Dice*- Index (Dice Similarity Coefficient – DSC) für manuell eingezeichnete Konturen und durch die DR generierte Konturen der Zielregionen mit dem Ergebnis einer rigiden Registrierung (RR) verglichen. Im Falle des Phantoms zeigten sich die stückweise rigiden Verfahren leicht überlegen, wobei sich featurelet_*NC*_ bei der intramodalen und featurelet_*MI*_ bei der intermodalen Registration auszeichneten. Im Fall der Prostata konnte nur in etwa 50 % der Fälle eine Verbesserung des DSC gegenüber der RR festgestellt werden. Es zeigte sich, dass *DR-Verfahren* das Ergebnis einer rigiden Registrierung im Fall der Lunge und auch in der Phantomstudie verbesserten, was im Fall der Prostata nicht signifikant nachgewiesen werden konnte. Eine eindeutig überlegene Methode zur DR konnte ebenfalls nicht ermittelt werden.

## 1 Introduction

Organ motion is a well known challenge in advanced conformal radiotherapy. The development and clinical introduction of radiation delivery units with integrated imaging option has stimulated research for the management and compensation of inter- and intrafractional patient movements, which is the primary goal of image guided adaptive radiotherapy (IGART) [1]. In general, the aim of IGART is a more precise dose delivery to the clinical target volume (CTV) and while at the same time reducing dose to organs at risk (OAR). Kilovoltage cone beam CT (CBCT) systems attached to conventional C-arm based linacs [2] and megavoltage fan beam CT as applied in tomotherapy units [3] represent today's most widely utilized volumetric imaging methods. In such a treatment concept deformable image registration (DR) is inevitable [4], [5], [6], [7], [8], [9], [10], [11]. Meanwhile, a number of commercial systems have been introduced to accomplish the task of deformable image registration [12], [13] for adaptive planning.

In general, a DR algorithm consists of (i) a rigid registration step, where translations and rotations are carried out for a gross alignment of the volume image data and if necessary also scaling is done and (ii) an algorithm to improve the match of the volume data content by defining a vector field that compensates for non-rigid motion of tissue [14]. Numerous methods were presented to determine such a vector field and systematic overviews can be found in literature [4], [10]. However, verification of the suitability of DR algorithms for clinical routine is scarce.

In this paper, we present a competitive validation of various non-rigid registration algorithms using a novel phantom setup and clinical data. In detail, a groupwise rigid registration algorithm [5] with different merit functions (normalized cross correlation and mutual information) was compared to a novel method based on the demons algorithm [8], [16] as implemented in the *iPlan* Software (BrainLAB AG, Feldkirchen, Germany). The validation took place using datasets of an especially designed deformable phantom. Intramodality and intermodality examples were studied as well as patients datasets from prostate and lung cancer consisting of planning CT and CBCT datasets acquired during the treatment course.

## 2 Materials and Methods

### 2.1 Piecewise deformable registration algorithm

An implementation of the featurelet-based deformable registration method suggested by Söhn et al. [5] was developed using the Insight Segmentation and Registration Toolkit (ITK, Kitware, Inc. New York, USA).

**Figure 1:**
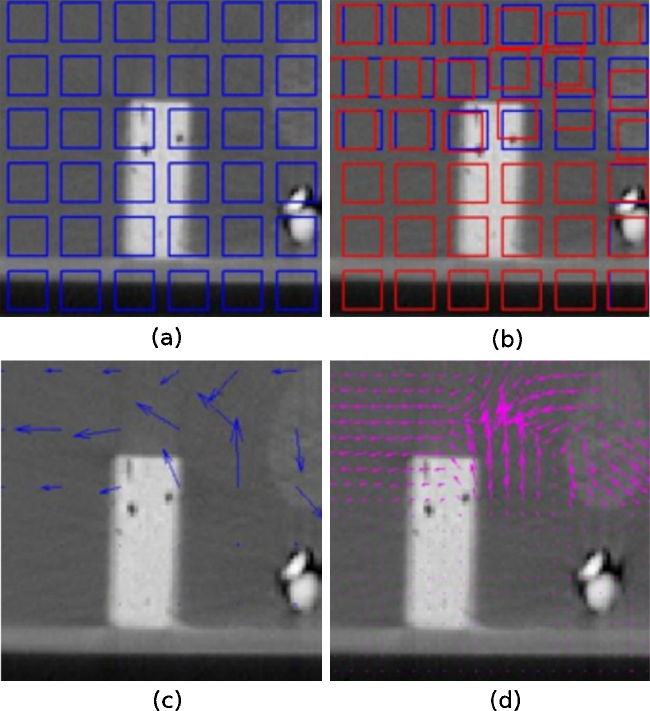
General steps of the piecewise non-rigid registration algorithm: (*a*) shows a representation of the volume divided in the subvolumes (featurelets) of regular size, (*b*) image shows the original position of the featurelets (blue) and the position after the registration process (red), (*c*) image shows the deformation field before interpolation, and on (*d*) the result of the interpolation can be found.

In the first step of the algorithm temporal subvolumes are created in both images. These are the featurelets (or megavoxels) of size A in the moving image – that is, the image that undergoes the spatial transform – and a search-region of size B defined on the reference image. In Fig. 1(a) a representation of the moving image divided in subvolumes of regular size can be seen; this figure was simplified for visualization, since in the algorithm all areas of the volume are covered by featurelets.

The featurelets of the moving image are then rigidly registered to its corresponding search-region on the reference image using a translation transform, a regular steepest gradient descent optimizer and either a Normalized Correlation Metric (NC) or a Mutual Information Metric (MI) to obtain the final displacement vectors of the megavoxels. Fig. 1(b) shows the original position of the featurelets (blue) and the position after the registration process (red).

Once the displacement vectors (Fig. 1(c)) were obtained for all the featurelets, the values of the transformation vectors were interpolated to all the voxels of the image by trilinear interpolation, and a restriction according to the final merit function value was imposed to avoid the misregistered featurelets to mislead the interpolation. The interpolated deformation field can be seen on Fig. 1(d). These interpolated vectors correspond to the deformation field.

Two of the three deformation fields where calculated using the piecewise deformable registration implementation using either of the two different metric functions referred above. These two methods will be referred as featurelet_*NC*_ and featurelet_*MI*_ respectively.

After a trial and error examination of the different parameters used for the registration, the optimized parameter set for all the cases were a featurelet size of 15 × 15 × 15 pixels, a search region of 30 × 30 × 30 pixels search-region, a maximum step length of 0.05 and a minimum of 0.001 for the gradient descent and a total of 2000 iterations.

### 2.2 *iPlan* adaptive algorithm

The Demons algorithm for deformable image registration was first presented by Thirion [17]. Since then it has been adapted, modified and compared to other algorithms [18], [19], [20]. The method is named “Demons” because in its original definition it is compared to the thermodynamics diffusion process, introducing a Maxwell-demon that regulates the diffusion using intensity differences and gradient information. The forces used are inspired from the optical flow equations and a smoothing process of the force vectors is done by Gaussian convolution. *iPlan* software uses a similar approach, initializing a grid of supporting points and optimizing in a global way for the whole object and not for subvolumes. According to the manufacturer, a global cross-correlation based measure is used for measuring image similarities here. The deformation fields for these algorithms were obtained on the *iPlan* treatment planning system (TPS). This method will be referred to as *iPlan* throughout this document. The deformable registration was done with the default parameters of the system and for the whole body.

### 2.3 Deformable Phantom, intermodality and intramodality registration

**Figure 2:**
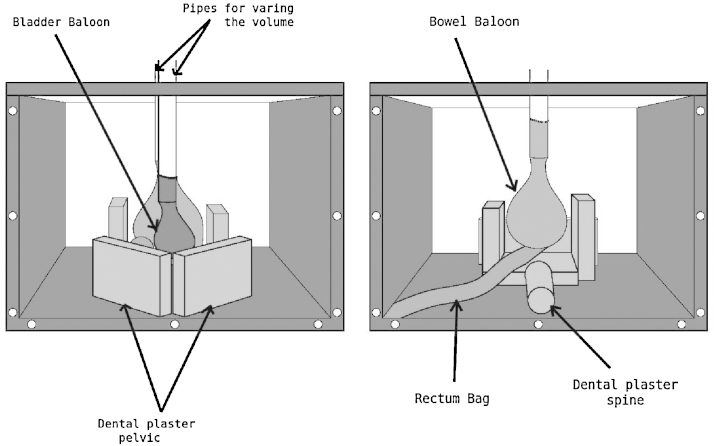
Pelvis-shaped deformable phantom. Dental plaster was used to represent the bony structures. Balloons were placed to simulate the bladder and intestines, a prostate-shaped polystyrene object (not visible in this figure) was glued to the bladder balloon and a plastic bag full of glass breads was used to simulate the colon. The size of the balloons was changed by injecting a water-iodine soap solution.

A purpose-built pelvis-shaped deformable phantom was designed. It consisted of a cubic plastic box of 40 × 40 × 29 cm^3^ with three opaque and three transparent walls, one of them being the removable top (Fig. 2). For simulation of intestine and bladder movement, two inflatable balloons were used and attached to the top of the phantom by means of two plastic pipes. The volume inside the balloon can be varied from 200 cc to 400 cc by injecting water with a syringe through these pipes. A prostate-shaped polystyrene object of approximately 110 cc was glued to the bladder balloon and tied to the colon by a plastic wire. This material was chosen since it is easy to contour and rigid. Six laminated radio-opaque pieces of dental plaster were glued to the bottom of the case to simulate the hip and the pelvic bones as well as the sacrum and the spine. The colon was simulated by a transparent plastic bag filled with glass beads. It was glued to the bottom of the box as well as to the above mentioned bony structures. The whole box was finally filled with water. The two balloons were filled with iodine soap solution, in different proportions, the bladder balloon being the one giving more contrast.

Three CT images (CT1, CT2 and CT3) of the phantom where acquired with a multislice CT scanner (Somatom Volume Zoom, Siemens, Erlangen, Germany, 120 kV, 200 mAs, 400 mm^2^ FOV and 4 mm slice spacing). For every acquisition the volume inside the balloons and the position of the structures was varied to obtain internal deformation. Then three CBCT images (XVI, Elekta, Crawley, United Kingdom) where also obtained changing the relative position of the structures inside the phantom (CT4, CT5 and CT6).

The six image datasets where imported in the *iPlan* (v.4.1, BrainLab, Feldkirchen, Germany) treatment planning system and rigidly registered. The bladder balloon, the prostate-shaped polystyrene item and the rectum-like bag where delineated manually on the six datasets. CT1 was defined as the planning CT and the other five used as the consecutive deformed datasets. The structures delineated on CT 1 where deformed using three different sets of the deformation fields obtained by the three algorithms per image set.

**Figure 3:**
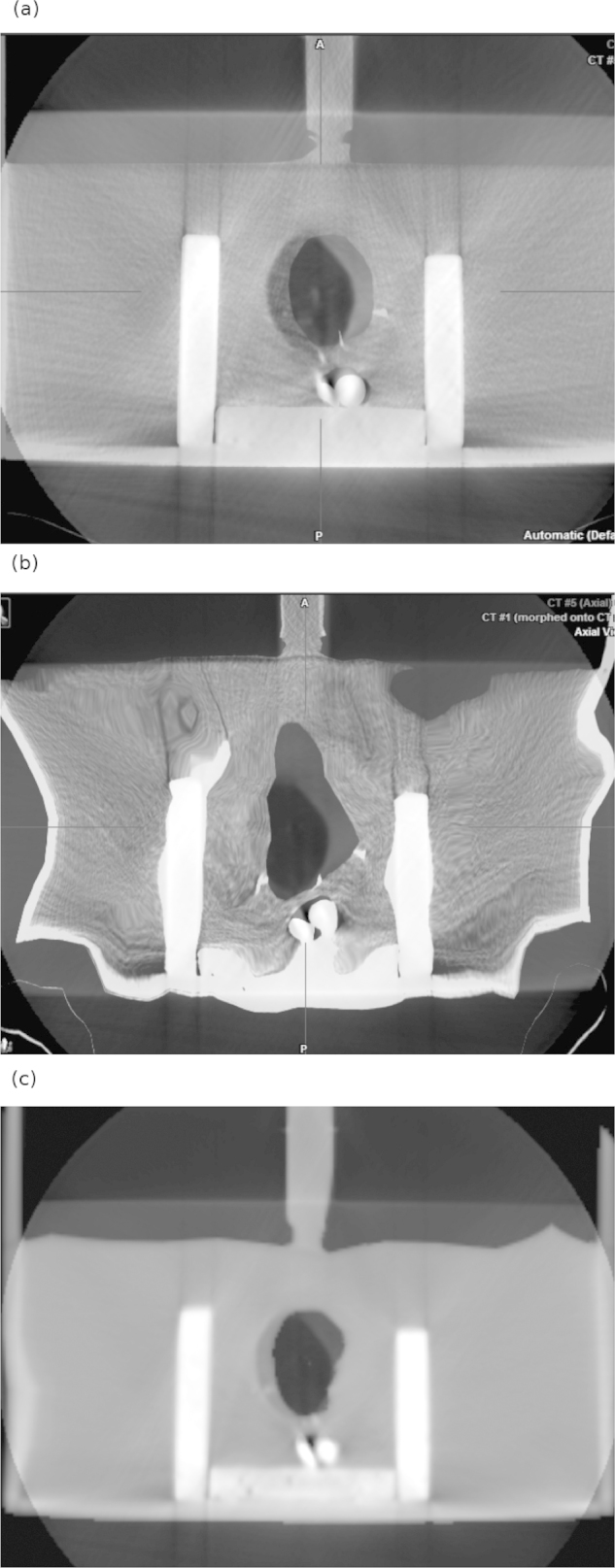
Pelvis-shaped deformable phantom (*a*). An overlay of CT1 (fixed image) and CT5 (CBCT image) after performing rigid registration (*b*). An overlay of CT1 (fixed image) and CT5 (CBCT image) after performing *iPlan* deformable registration (*b*) An overlay of CT1 (fixed Image) and CT5 (CBCT image) after performing featurelet_*MI*_ deformable registration.

In Fig. 3(*a*) one of the internal deformations of the phantom can be found; here, CT1 and CT5 are overlaid and the displacement of the prostate-like structure can be clearly seen. Also, the pseudo pelvic and rectal structures are apparently coincident. Fig. 1(*b*) and Fig. 1(*c*) correspond to the overlap after performing deformable registration, for *iPlan* method and featurelet_*MI*_ respectively. It can be observed that the featurelet_*MI*_ method is deforming the central prostate-shaped structure but is not bending the box structure as much as the *iPlan* method.

### 2.4 Prostate Cases, intermodality registration

The images of nine patients treated for prostate cancer were arbitrarily selected for this study. The dataset consisted of one planning CT acquired with a multislice CT scanner (Somatom Volume Zoom, Siemens, Erlangen, Germany) using 120 kV, 200 mAs and 4 mm slice spacing. Furthermore, 7 weekly CBCTs (XVI, Elekta, Crawley, United Kingdom) were taken for all cases, resulting in 63 CBCT scans. Acquisition parameters were chosen according to recommended prostate protocol without bow-tie filter and an axial field of view (FOV) of 42 cm and 12 cm scan length. The reconstructed volume was converted to 4 mm slice thickness. Every CBCT was rigidly registered to the planning CT using the *iPlan* Image Fusion application. Prostate, rectum and bladder were delineated on the panning CT and on all CBCTs of all data set by one radiation oncologist on the same treatment planning system. All structures defined on the CBCTs were mapped to the planning CT. The contours drawn on the planning CT were deformed with respect to the CBCT images; the deformation fields were obtained by a DR between the reference CT and the correspondent CBCTs.

### 2.5 Lung Cases, intermodality registration

Ten arbitrarily selected patients undergoing stereotactic body radiation therapy (SBRT) for non-small cell lung cancer (NSCLC) or lung metastasis were selected for this study. Image acquisition was done with patients positioned in the BodyFIX system (Medical Intelligence/Elekta, Schwabmünchen, Germany) for imaging and treatment. CT and CBCT imaging was performed under free-breathing conditions.

Treatment planning CT images were again acquired with a Siemens Somatom Volume Zoom CT scanner (120 kV, 120 mAs and 4 mm slice thickness) and with intravenous contrast (Japomiro, Bracco, Vienna, Austria, 90 ml). CBCT images (XVI, Elekta, Crawley, United Kingdom) were obtained without contrast before each treatment fraction on the linear accelerator. The CBCT acquisition protocol (120 kV, 649 mAs) was optimized for thorax imaging with a field of view of 42 cm. The duration of CBCT acquisition was approximately 2 minutes, whereas CT imaging was performed in 15 seconds. The reconstructed volume from CBCT was converted to 4 mm slices and transferred to the treatment planning system *iPlan*, which was used for contouring.

For each patient, the treatment planning CT and one randomly selected CBCT set (out of three available) were chosen for analysis. The gross target volume (GTV) was delineated in both the CT and the CBCT images of all the ten cases. The DR methods were applied to the GTV contour of the CT to establish a correspondence to the CBCT.

### 2.6 Evaluation

For analysing all the contours obtained by the different deformation parameters of the CT delineations on the three different dataset groups, the Dice similarity coefficient (DSC), which is sometimes also named volume overlap index (VOI), was used. This index is defined as:

(1) $$\mathrm{DSC}=\frac{V_{d}\cap V_{m}}{(V_{d}\cup V_{m})/2}\times 100$$

where *V*_*d*_ is the deformed volume and *V*_*m*_ is the reference volume. In our case *V*_*m*_ corresponds to the volume obtained from the contour manually drawn on the CBCTs and *V*_*d*_ corresponds to the volumes obtained by deforming the contours from the CT.

As a second tool for performance assessment of the registration methods the *Hausdorff distance*

(2) H(A,B)=max(h(A,B),h(B,A)

where

(3) $$h(A,B)=\max_{a\in A}\min_{b\in B}∥a-b∥$$

was also calculated for the contours of the organs of interest before and after deformable registration. The Hausdorff-distance gives the maximum distance in pixels between two contours performing the calculation to the nearest point in both directions, from contour A to B and vice versa.

For all test conditions the starting point for making the comparisons is the rigid registration performed in *iPlan* (RR).

The deformable phantom was used to analyse the performance of the algorithms in two different deformation scenarios; mainly, we were looking to achieve a better contouring precision than in clinical images due to the high contrast of the phantom images in both CT and CBCT acquisitions. For the intramodality registration analysis of the deformable phantom, CT1 was considered to be the reference (or fixed) image and CT2 and CT3 the moving images. After performing the rigid and deformable registrations between these images the DSC and the Hausdorff-distance were quantified. Statististical significance was determined using a Wilcoxon signed rank test.

Both validations are critical for the quantification of contour propagation, which is an essential tool for the assessment of the total dose delivered.

## 3 Results

### 3.1 Deformable Phantom, intermodality and intramodality registration

**Figure 4:**
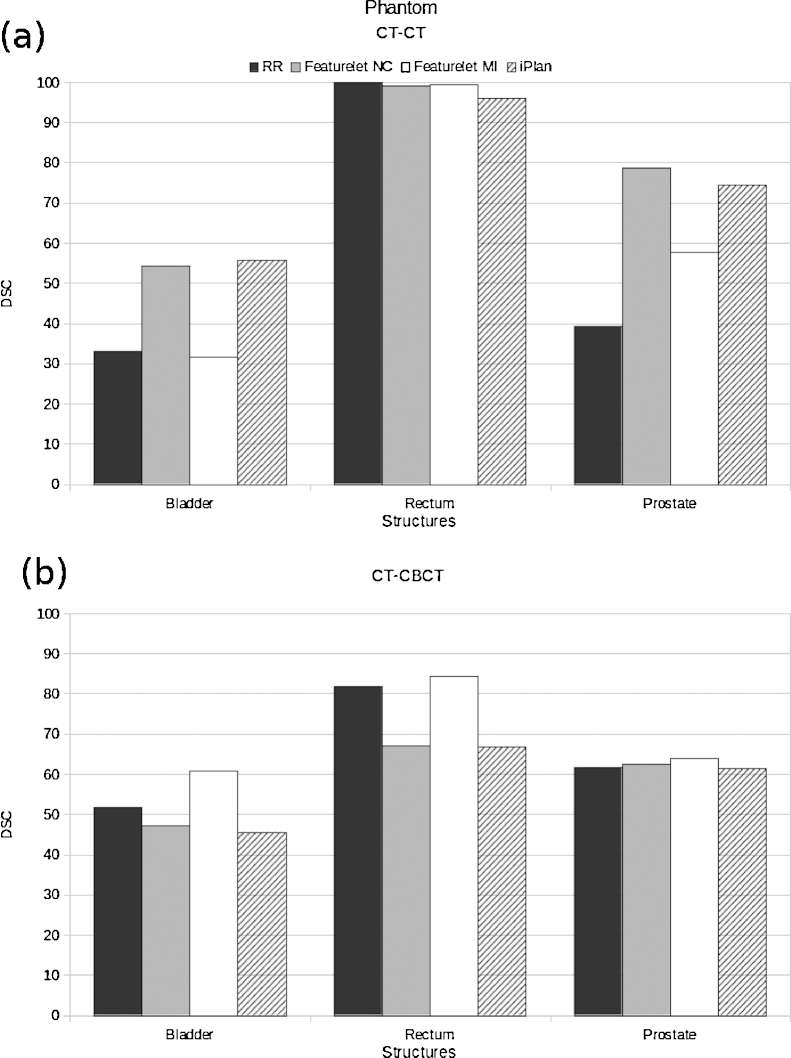
(*a*) DSC values for the phantom CT-CT deformable registration; here, two deformations are included. It can be seen that on average the featurelet_*NC*_ method and the *iPlan* method had a similar performance. The featurelet_*MI*_ method is not improving the DSC substantially. (*b*) DSC values for the phantom CT-CBCT deformable registration. Here, three deformations are included. For both bladder and rectum, the featurelet_*NC*_ and *iPlan* methods are reducing the DSC value, and almost not changing it for the prostate. On the other hand, for the three structures the featurelet_*MI*_ method is improving the DSC. RR corresponds to the rigid registration starting point, featurelet_*NC*_ to the featurelet deformable registration method using normalized correlation metric, featurelet_*MI*_ to the featurelet deformable registration method using mutual information metric and *iPlan* corresponds to the deformable registration performed using the *iPlan* -adaptive application.

The results of the average DSC for the phantom studies can be found in Figure 4. For the first structure studied, the bladder, the reference value of the DSC after RR was 33.1. It was found that the featurelet_*MI*_ method was not changing the average DSC value for this case, keeping it under 35. The featurelet_*NC*_ and the *iPlan* method were increasing the coefficient to 52 and 54, respectively. For the rectum the reference DSC is 100 because the structure is not moving at all and the rigid registration is performed in such a way that the coincidence of the bone in both images is fully achieved. For the three methods the value was slightly decreasing down to 96 for the *iPlan* method. For the prostate the initial or reference average DSC was 39.4, and had an considerable increase to 78.6, 57.8 and 74.5 for the featurelet_*NC*_, featurelet_*MI*_ and *iPlan* methods respectively.

For the intermodality cases, CT1 was considered to be the reference or fixed image and CT4, CT5 and CT6 the moving images that correspond to the three CBCTs acquired in the treatment room. For all the structures only the featurelet_*MI*_ was improving the DSC, showing a considerable improvement for the bladder, going from a 51.8 to 60.8.

**Figure 5:**
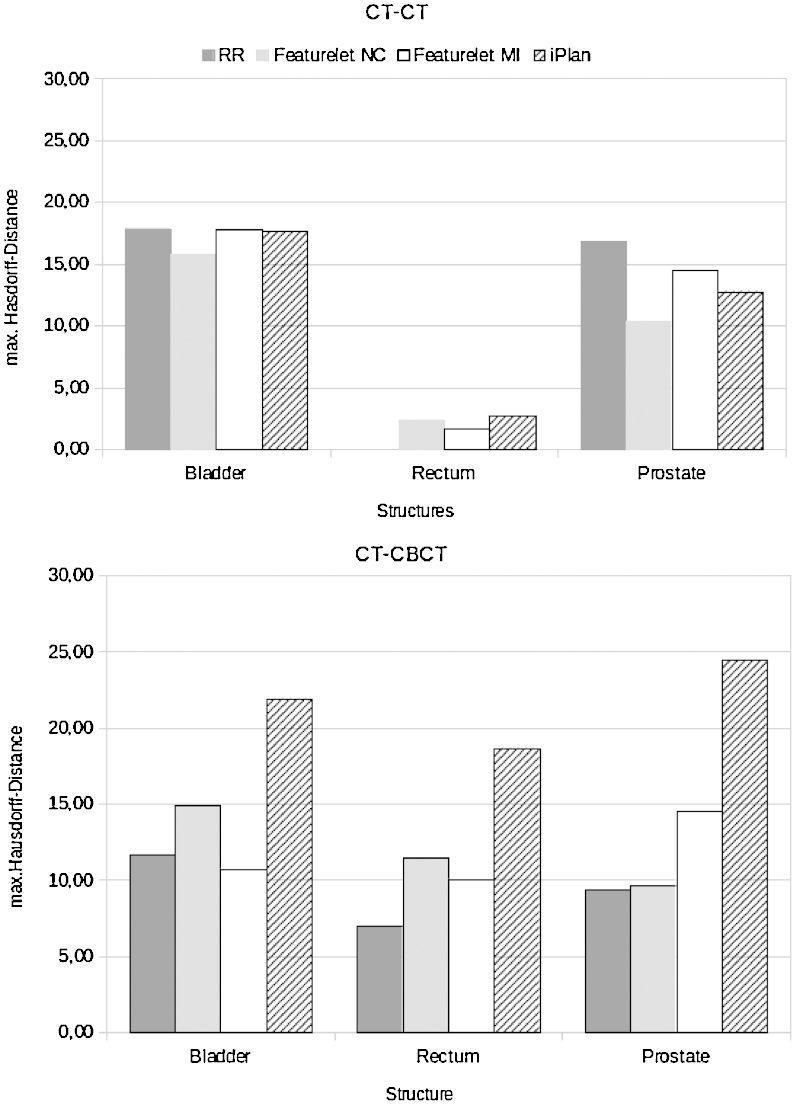
(*a*) Hausdorff distance values for the phantom CT-CT deformable registration. Here two deformations are included. It can be seen that on average the featurelet_*NC*_ method reveals best performance on the bladder and the prostate structures. The featurelet_*MI*_ method is not improving the registration result substantially and the *iPlan* method only improve the *Hausdorff-distance* values for the prostate structure. (*b*) Hausdorff distances for the phantom CT-CBCT deformable registration, here three deformations are included, it can be observed that for all the structures no method improves the Hausdorff distance in comparison to the rigid registration. RR corresponds to the rigid registration starting point, featurelet_*NC*_ to the featurelet deformable registration method using normalized correlation metric, featurelet_*MI*_ to the featurelet deformable registration method using mutual information metric and *iPlan* corresponds to the deformable registration performed using the *iPlan* -adaptive application.

The results of the average Hausdorff-distance for the phantom studies can be found in Figure 5. For the first structure studied, the bladder, the reference average value of the Hausdorff-distance after RR was 17.83 pixels. The featurelet_*MI*_ method as well as the *iPlan* method were not changing the average Hausdorff-distance value in this case. The featurelet_*NC*_ was decreasing the distance to 15.74 pixels. For the rectum the reference Hausdorff-distance was 0 pixels since the structure is not moving at all and the rigid registration is performed in such a way that the coincidence of the bone of both images is fully achieved. For the three methods the value was increasing up to 2.67 pixels for the *iPlan* method. For the prostate the initial Hausdorff-distance was 16.8, and had a considerable decrease to 10.37, 14.49 and 12.77 pixels for the featurelet_*NC*_, featurelet_*MI*_ and *iPlan* methods respectively.

The prostate type polystyrene object did not vary its shape or size so also a study of the volume in voxels and the position of the center of mass was done. The *iPlan* method was the one modifying the volume of the prostate to the largest extent which resulted on average change of the volume of 35%. The featurelet_*NC*_ method was changing it by 10% and the featurelet_*MI*_ method by less than 5%. The displacement of the center of mass of the prostate volume for the CT-CT images was 47.7 ± 14.7 mm on average. After featurelet_*NC*_ deformable registration it was 17.3 ± 12.4 mm, after featurelet_*MI*_ it was 37.1 ± 22.4 mm and after *iPlan* it was 20.6 ± 3.6 mm. For the CT-CBCT cases the original displacement on average was 26 ± 12.9 mm, and 23.1 ± 10, 24.2 ± 10.7 and 29.8 ± 10.2 mm after featurelet_*NC*_, featurelet_*MI*_ and *iPlan* registration respectively.

### 3.2 Prostate Cases, intermodality registration

The featurelet_*NC*_ method was deforming the structures in a way that no improvement over the initial RR could be found. (*a*) The featurelet_*MI*_ and *iPlan* methods are not significantly different from the result of RR for the rectum. (*b*) For the prostate on average all of the deformable registration methods show a deterioration of registration results compared to RR. (*c*) For the bladder, the featurelet_*MI*_ method is not significantly different compared to the RR but the *iPlan* method is significantly improving the Hausdorff distance.

**Figure 6:**
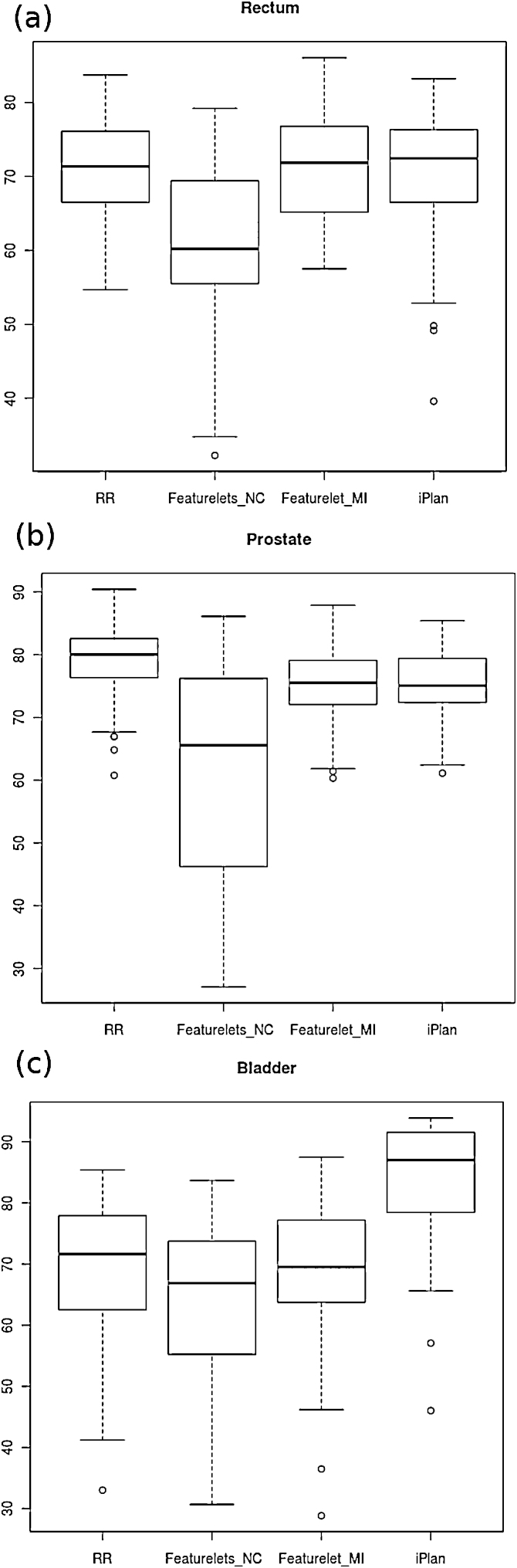
Boxplot of the DSC for the rectum, prostate and bladder in the prostate patient cases. The featurelet_*NC*_ method did not work at all, therefore no improvement in registration was observed. (*a*) The featurelet_*MI*_ and *iPlan* methods are not significantly different than the RR for the rectum. (*b*) For the prostate on average all of the deformable registration methods are worst than the starting point of the RR (*c*) For the bladder, the featurelet_*MI*_ method is not significantly different than the RR but the *iPlan* method is significantly improving the DSC.

For the clinical cases of prostate patients, 62 deformations were analysed. The results obtained for the DSC of the three structures studied can be observed on the boxplots on Figure 6. For the rectum, Figure 6(a), the featurelet_*NC*_ method was on average significantly worse than the original DSC value after doing RR and the other methods were not changing the result considerably – only an increase of outliers was achieved after performing the deformable registration of *iPlan*. For the prostate all the contours generated by the three deformations gave a worse DSC value than the RR (Fig. 6 (b)). Only on the bladder the deformation method was an improvement in comparison to the RR reaching an average DSC for the *iPlan* method of over 85 (Fig. 6c). The results of the Wilcoxon signed rank test for the DSC and Hausdorff distance using the three methods for the deformations on the rectum, bladder and prostate of the clinical prostate cases can be found in Table 1. Only the result achieved with the *iPlan* method for the DSC of the bladder contour was significantly better than the RR and the featurelet_*MI*_.

**Table 1:** Wilcoxon signed rank test for the DSC and the Hausdorff distance using different methods for the deformations on the three main structures of the prostate cases.

Structure	Method 1	Method 2	Significance DSC	Significance Hausdorff
Bladder	RR	featurelet_*NC*_	–	NS
Bladder	RR	featurelet_*MI*_	NS	NS
Bladder	RR	*iPlan*	++	++
Bladder	featurelet_*MI*_	*iPlan*	++	++
Rectum	RR	featurelet_*NC*_	–	–
Rectum	RR	featurelet_*MI*_	NS	NS
Rectum	RR	*iPlan*	NS	NS
Rectum	featurelet_*MI*_	*iPlan*	NS	NS
Prostate	RR	featurelet_*NC*_	–	–
Prostate	RR	featurelet_*MI*_	–	NS
Prostate	RR	*iPlan*	–	–
Prostate	featurelet_*MI*_	*iPlan*	NS	NS

The abbreviation NS stands for method 2 is not significantly different to method 1, + or ++ if method 2 is significantly better than method 1 and – if method 2 is significantly worse than method 1.

**Figure 7:**
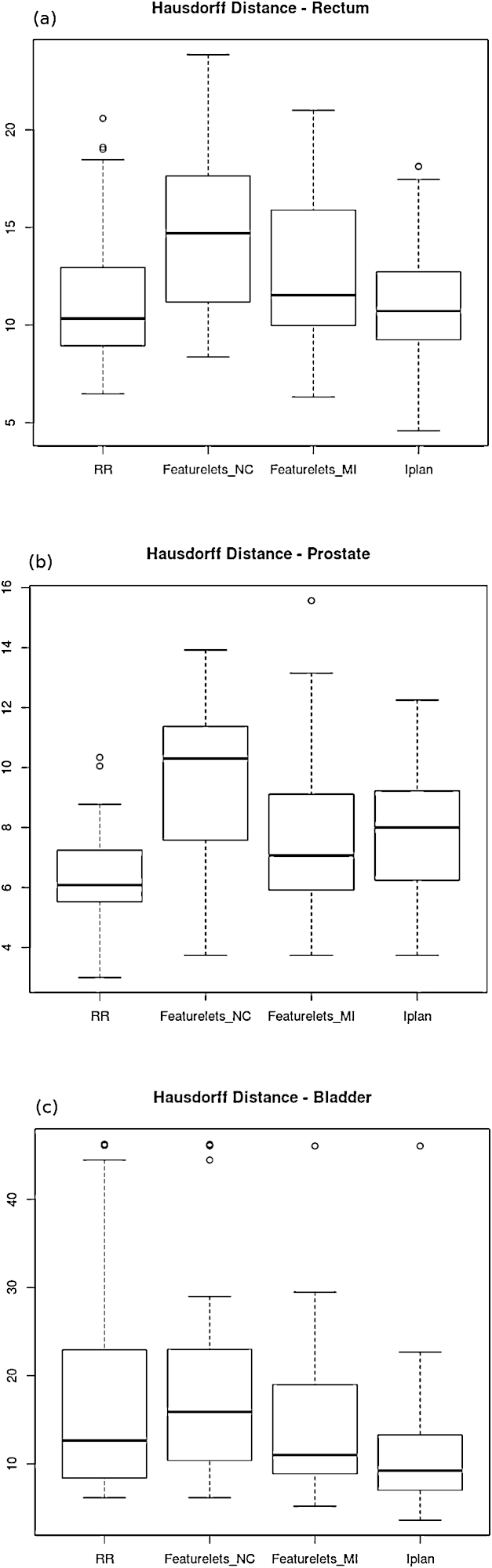
Boxplot of the *Hausdorff-distance* for the rectum, prostate and bladder in the prostate patient cases.

The results obtained for the Hausdorff-distance of the three structures studied can be observed on the boxplots on Fig. 7. For the rectum (Fig. 7 (a)) the featurelet_*NC*_ method was on average significantly worse than the original Hausdorff Distance value after doing RR. The other methods were not significantly worse, but they were not an improvement. For the prostate all the contours generated by the three deformations gave a worse Hausdorff-distance value than the RR (Fig. 7 (b)). Only in the case of the bladder the deformation method was an improvement in comparison to the RR reaching a average Hausdorff distance for the *iPlan* method of 10.38 pixels in comparison to 17.64 pixels for the original RR (Fig. 7 (c)). Only the improvement obtained with the *iPlan* method for the Hausdorff-distance of the bladder contour was significantly better than the RR and the featurelet_*MI*_.

### 3.3 Lung Cases, intermodality registration

**Figure 8:**
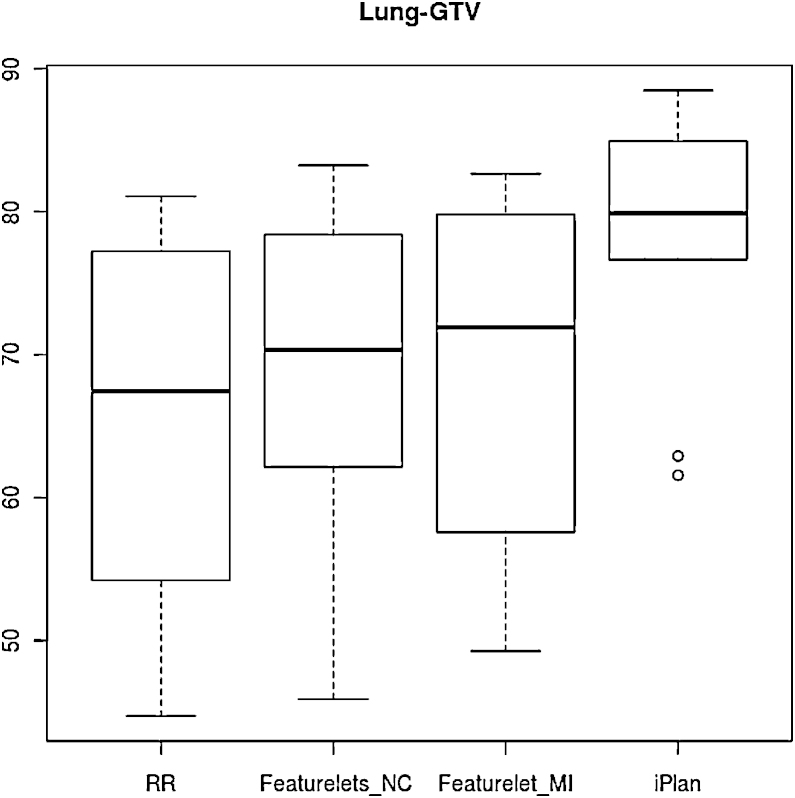
Boxplot of the dice similarity coefficient for the 10 cases of lung GTV for rigid registration RR, piecewise normal correlation featurelet_*NC*_ and mutual information featurelet_*MI*_ as well as the *iPlan* deformation method. All methods exhibit a significant improvement compared to RR, but *iPlan* outperforms other methods significantly.

In the clinical lung cases all the methods gave a significant improvement on the DSC for GTV in comparison to the RR. The maximum improvement was for the *iPlan* method which was also significantly better than the other two methods. Figure 8 illustrates that the *iPlan* method achieved the highest mean DSC and the smallest result range. In table 2 the results of the Wilcoxon signed rank test for the DSC of the GTV volume using different methods of deformation are shown.

**Table 2:** Wilcoxon signed rank test for the DSC and the Hausdorff distance using different methods of deformation on the lung cases.

Method 1	Method 2	Significance DSC	Significance Hausdorff
RR	featurelet_*NC*_	+	+
RR	featurelet_*MI*_	++	NS
RR	*iPlan*	+	NS
featurelet_*MI*_	*iPlan*	+	NS
featurelet_*NC*_	featurelet_*MI*_	NS	NS

The abbreviation NS stands for method 2 is not significantly different to method 1, + or ++ if method 2 is significantly better than method 1 and – if method 2 is significantly worse than method 1.

**Figure 9:**
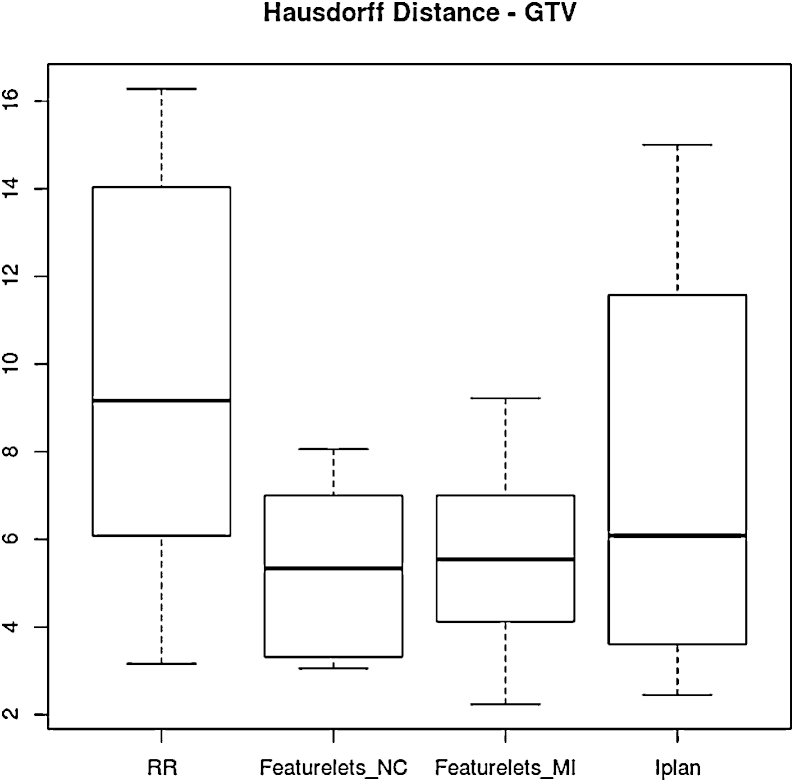
Boxplot of the *Hausdorff Distance* for the 10 cases of lung GTV for rigid registration RR, piecewise normal correlation featurelet_*NC*_ and mutual information featurelet_*MI*_ as well as the *iPlan* deformation method. All methods are an improvement from rigid registration method, but only the featurelet_*NC*_ method was statistically significantly better then the RR starting point.

For the Hausdorff-distance only the the featurelet_*NC*_ method was significantly better then the RR starting point although is evident from Figure 9 that all the methods achieve an improvement over RR.

## 4 Discussion

The topic of DIR has gained importance in radiation oncology since it is generally considered as being a prerequisite for ART. For example it allows performing contour propagation in a time efficient manner, since it eliminates the need for workload intensive manual contouring. Furthermore DIR is needed for dose accumulation in adaptive approaches. Dose accumulation itself, although an important research field in the medical physics community, is basically a badly needed tool for further development of radiation oncology. This tool allows tracking the dose during the course of radiotherapy, which can be severely affected by anatomic variations. Of course tracking the tumor dose is of importance, but tracking the doses in organs at risk is (at least) of the same importance. In most advanced radiotherapy approaches tolerance doses to OAR drive the computerized optimization approaches in treatment planning. The current knowledge on tolerance doses for organs at risk is based on static images, volumes defined at the time of treatment planning from these static images, dose volume relations extracted from that information, which is in the final stage related to observed toxicity. This methodological approach is the basis for data in the recently published QUANTEC report [24]. In order improve this current radiobiological knowledge and dataset, respectively, dose accumulation is needed to get a better estimation of the “true” doses to OARs. Volumetric imaging tools for IGRT, such as kV or MV CT, deliver the imaging basis for ART approaches. Although dose accumulation is not the primary focus of the present study, it is the main motivation for research on DIR in our group.

The number of recently published papers on DIR and dose accumulation is considerable [25], [26], [27]. Most of them focus either on the presentation of the algorithm or their application for certain pathologies without having a real ground truth information for benchmarking the respective approach [28], [29]. In other words very little information has been published on validation of DIR. One example was recently presented in literature using a two dimensional deformable phantom with a balloon catheter to simulate tumor growth for head and neck cancer patient [30]. The two-dimensional approach had the advantage that by the use of a camera and nonradiopaque markers no influence on the deformation algorithms could be expected and they could be independently benchmarked. On the other hand they stated that the phantom would benefit from more electron density heterogeneity. This and a three-dimensional extension was actually what we were aiming for with our purpose-built prostate phantom presented in this study. As the phantom can be easily imaged in different filling conditions this is a DIR validation approach that provides inherently ground truth information. This phantom is mimicking the pelvic anatomy with flexible structures and certainly not a general-purpose phantom. For all cases the rectum was not changing in neither position nor size, which is certainly an over simplification. The prostate was modified just in position, and the bladder was changing in position, shape and size. The design of a pelvic DIR verification phantom was motivated by current activities to implement ART for pelvic malignancies.

However, one potential application still requiring more detailed examination on the usefulness of DIR is lung motion; while tracking approaches using local rigid registration do exist [11], it is to evaluated separately whether the methods presented in this paper are applicable to the same extent for lung irradiation. The results presented here do not take into account intrafractional motion. Therefore, additional validation on dynamic image data is necessary.

Beside phantom based validation of deformable registration other approaches e.g. landmarks in multiple datasets like 4D-CT for lung and liver annotated by a physician are used [10]. Such point based estimations of registration errors can be used to benchmark algorithms in a multi-institutional setting although no volume information is available. In addition the result of a deformable image registration, namely the deformation vector field and its “physical characteristics” is of interest for various research groups [31], [32]. Measures which are applied are for example inverse consistency error, the Jacobian or harmonic energy.

For our phantom study, we divided the evaluation into two main groups, the intra-modality and the inter-modality. Three volumes were analysed in both groups, the bladder, rectum and prostate. For the first phantom group (see also Fig. 2) using imaging information for DIR from same imaging equipment (intra-modality) results for MI method were the worst, although it was not generating errors. The NC method and the *iPlan* method had a very similar performance, although NC was slightly better. On the other hand the results in Figure 4 indicate that for inter-modality DIR the MI is having the best performance for all the structures.

It is a well known fact that for prostate cases the image contrast is very poor in either CT and CBCT modalities, efforts for auto-segmenting structures in this treatment area have been done and studied [21]. For analyzing our data we decide to use the DSC that evaluates the behavior of the deformable registration concerning a hole volume, instead of the target registration error (TRE) that estimate the position of just specific points in the body. It has been also shown that the inter-observer variability for target volume delineation in prostate cancer is larger for CBCT-based contouring [16] In our study the deformed contour obtain by using the deformation field on the contours drawn on the CT were also compared to the ones drawn directly on the CBCT image, so, it is logical to assume that both are not a 100% accurate delineation, specially compared to the one done on the phantom. In a recent study Thor et al. [23] used a similar approach, where automated contours where compared with manual delineations. An improvement of the DSC of 3 (prostate), 6 (rectum) and 9 (bladder) points for the five patients with a total of 36 scans was reported. These results are comparable to the 4, 2, and 3 points increase obtained in our study with thefeaturelet_*MI*_ algorithm, and the 6, 5 and 17 points increase for the *iPlan* method in our study.

As mentioned before for the clinical lung case with all the deformable registration methods a significant improvement was achieved on the DSC (see Table 1).This suggests that the main issue for improving algorithm performance is not only the ability of the algorithms to operate on different image modalities, but in different treatment locations, this was also reaffirmed by the Hausdorff Distance analysis. Future developments on the featurelet algorithm will focus on the refinement of the search region as well as using image gradients for the selection of “more important” featurelets and proper interpolation of the deformation vector field in between.

## 5 Conclusions

In this work we presented an analysis of 3 deformable registration algorithms In general, featurelet algorithms that are based on piecewise registration methods were found to be comparable to the Demons algorithm implemented in the *iPlan* -adaptive software. Despite the fact that very good results for deformable registration on phantoms and clinical data have been reported [5], [15] (mainly for 4D respiratory CTs and externally deformed regular shaped phantoms), we found no clear superiority of any method in the clinical cases for the prostate; for lung cases the *iPlan* method performed best.
